# Accuracy of Apple Watch Measurements for Heart Rate and Energy Expenditure in Patients With Cardiovascular Disease: Cross-Sectional Study

**DOI:** 10.2196/11889

**Published:** 2019-03-19

**Authors:** Maarten Falter, Werner Budts, Kaatje Goetschalckx, Véronique Cornelissen, Roselien Buys

**Affiliations:** 1 Cardiology Department University Hospitals Leuven Leuven Belgium; 2 Department of Cardiovascular Sciences KU Leuven Leuven Belgium; 3 Department of Rehabilitation Sciences KU Leuven Leuven Belgium

**Keywords:** mobile health, heart rate, energy expenditure, validation, Apple Watch, wrist-worn devices, cardiovascular rehabilitation

## Abstract

**Background:**

Wrist-worn tracking devices such as the Apple Watch are becoming more integrated in health care. However, validation studies of these consumer devices remain scarce.

**Objectives:**

This study aimed to assess if mobile health technology can be used for monitoring home-based exercise in future cardiac rehabilitation programs. The purpose was to determine the accuracy of the Apple Watch in measuring heart rate (HR) and estimating energy expenditure (EE) during a cardiopulmonary exercise test (CPET) in patients with cardiovascular disease.

**Methods:**

Forty patients (mean age 61.9 [SD 15.2] yrs, 80% male) with cardiovascular disease (70% ischemic, 22.5% valvular, 7.5% other) completed a graded maximal CPET on a cycle ergometer while wearing an Apple Watch. A 12-lead electrocardiogram (ECG) was used to measure HR; indirect calorimetry was used for EE. HR was analyzed at three levels of intensity (seated rest, HR1; moderate intensity, HR2; maximal performance, HR3) for 30 seconds. The EE of the entire test was used. Bias or mean difference (MD), standard deviation of difference (SDD), limits of agreement (LoA), mean absolute error (MAE), mean absolute percentage error (MAPE), and intraclass correlation coefficients (ICCs) were calculated. Bland-Altman plots and scatterplots were constructed.

**Results:**

SDD for HR1, HR2, and HR3 was 12.4, 16.2, and 12.0 bpm, respectively. Bias and LoA (lower, upper LoA) were 3.61 (–20.74, 27.96) for HR1, 0.91 (–30.82, 32.63) for HR2, and –1.82 (–25.27, 21.63) for HR3. MAE was 6.34 for HR1, 7.55 for HR2, and 6.90 for HR3. MAPE was 10.69% for HR1, 9.20% for HR2, and 6.33% for HR3. ICC was 0.729 (*P*<.001) for HR1, 0.828 (*P*<.001) for HR2, and 0.958 (*P*<.001) for HR3. Bland-Altman plots and scatterplots showed good correlation without systematic error when comparing Apple Watch with ECG measurements. SDD for EE was 17.5 kcal. Bias and LoA were 30.47 (–3.80, 64.74). MAE was 30.77; MAPE was 114.72%. ICC for EE was 0.797 (*P*<.001). The Bland-Altman plot and a scatterplot directly comparing Apple Watch and indirect calorimetry showed systematic bias with an overestimation of EE by the Apple Watch.

**Conclusions:**

In patients with cardiovascular disease, the Apple Watch measures HR with clinically acceptable accuracy during exercise. If confirmed, it might be considered safe to incorporate the Apple Watch in HR-guided training programs in the setting of cardiac rehabilitation. At this moment, however, it is too early to recommend the Apple Watch for cardiac rehabilitation. Also, the Apple Watch systematically overestimates EE in this group of patients. Caution might therefore be warranted when using the Apple Watch for measuring EE.

## Introduction

Mobile health has been growing tremendously in the last decade. Future perspectives are promising for further growth and integration of mobile technology in health care. One type of technology that is particularly interesting for mobile health is the wrist-worn device capable of monitoring a large variety of parameters including heart rate (HR), energy expenditure (EE), steps taken, distance traveled, and in the near future possibly even oxygen saturation, blood glucose, and cardiac arrhythmia [1-2]. Demand in patient population is also rising, with recent studies showing that up to one-third of patients with chronic heart disease use personal heart rate monitors and over two-thirds of patients who don’t already use a heart monitor reporting that they appreciate heart monitoring as being important for home-based exercise [3].

Wrist-worn devices have the ability to monitor vital parameters and provide the user with an overview and feedback on the collected data. Validation studies comparing assessments by these devices to clinically approved measurements are often lacking. The Apple Watch uses photoplethysmography (PPG) with optical sensors at the wrist to measure HR. EE is calculated with algorithms that are not openly disclosed [4].

Validation studies have been done to evaluate the accuracy of HR, EE, and other measurements in healthy subjects for a variety of fitness trackers [4-16]. Boudreaux et al [6] tested eight devices for accuracy of HR and EE measurements on healthy subjects and found that HR accuracy from wearable devices differed at different exercise intensities with an increasing underestimation of HR at higher exercise intensities. It was also found that EE estimates were inaccurate. They conclude that wearable devices are not medical devices and users should be cautious when interpreting results of activity monitoring. Shcherbina et al [12] tested seven devices on healthy subjects and found that HR measurements were within acceptable error range (5%). However, none of the tested devices had EE estimates within an acceptable range.

Modern health care is shifting its focus to home-centered health care with the aid of mobile technology. This study aimed to assess if commercially available mobile health technology such as the Apple Watch could be used for monitoring home-based exercise in future cardiac rehabilitation programs. The purpose of this study was to evaluate the accuracy of the Apple Watch with regard to HR and EE measurements during exercise in patients with cardiovascular diseases.

## Methods

### Ethics

This study was conducted in accordance with the declaration of Helsinki and approved by the local institutional review board (registration number S58592). A written informed consent was obtained from every patient before inclusion in the study.

### Patient Recruitment

Patients were recruited at the cardiovascular rehabilitation consultation of the University Hospitals Leuven (Leuven, Belgium). All patients scheduled for a cardiopulmonary exercise test (CPET) as part of their cardiovascular rehabilitation program were consecutively included; one patient was excluded due to inability to use the VO_2_ mask due to recent laryngeal surgery. Patients were equipped with the Apple Watch during their CPET.

The participant number of 40 patients was determined based on the results of Wallen et al [4] considering a power of 0.5 and probability of type I error of 5%. This sample size is in line with comparable studies [4-7,10,12] of wrist-worn health-tracking devices where participant numbers ranged from 20 to 60 patients.

### Device and Data Collection

The Apple Watch (Apple Inc) is a wrist-worn commercially available device that uses PPG for HR assessment. For this study, the Apple Watch Sport 42 mm (first generation) was used. The device was bought commercially and handled according to the manufacturer’s instructions.

The device was attached to the patient’s left wrist. Weight and height of the patient were recorded in the iPhone Health app before the test was started. On the Apple Watch Workout app, the option Indoor Cycling was chosen. On this app, the workout was started at the beginning of the resting phase of the CPET. Registrations were stopped at the same cutoff point as the stopping of the CPET because of patient exhaustion (cycling <60 rotations per minute).

Data were extracted using the iPhone Health app and the iPhone Health Export app. The Health app provided HR at 5 second intervals and EE at 2 to 3 second intervals. HR was converted to mean HR per 30 seconds; EE was analyzed as cumulative EE over the duration of the CPET test.

Other information collected included demographic data (gender, age, and anthropometrics: weight, height, body mass index [BMI]), peak oxygen uptake (peak VO_2_), VO_2_, and carbon dioxide (VCO_2_). The heart rate reserve (HRR) of each patient was calculated as the difference between the maximum and minimum HR as measured by electrocardiogram (ECG).

### Exercise Protocol

Patients performed a CPET test in normal conditions, having eaten and taken their routine medication, often including a beta-blocker. During this exercise test, participants wore the Apple Watch on their left wrist and wore a metabolic system (Jaeger Oxycon, Vyaire Medical Inc) for breath oxygen uptake and carbon dioxide output measurements and a 12-lead ECG (Cardiosoft, General Electric Company) for recording HR and heart rhythm. During the CPET, the ECG was constantly monitored by one of the researchers for cardiac arrhythmia. All tests were performed in a laboratory setting at a controlled room temperature of 21°C to 23°C.

The CPET started with 1 minute of seated rest. The exercise then started at 20 watts and load was increased with 20 W/min [17]. This protocol was adjusted to a faster or slower increase in cycling resistance depending on physical fitness and based on previous CPET records.

### Statistical Analysis

Descriptive data are reported as mean and standard deviation or as median and range. Gas analysis data from indirect calorimetry (VO_2_ and VCO_2_) served as criterion measurement for calculations of EE (kilocalories per minute). For conversion of VO_2_ and VCO_2_ to caloric expenditure (kcal), the Weir equation [18] was used: kcal/min = ([1.1xRQ]+3.9)xVO_2_.

Twelve-lead ECG was used as criterion measurement for HR (beats per minute).

For analysis purposes, HR was analyzed for three 30 second intervals: one interval at the initial 30 second of the test (seated rest, HR1), one in the middle of the CPET time (moderate intensity based on test duration, HR2), and one interval prior to and including maximal performance level (HR3). EE was compared for each patient for the entire duration of the test.

Mean difference (MD) and standard deviation of the mean difference (SDD) were calculated. MDs were tested for normality using the Shapiro-Wilk test. Bland-Altman plots were constructed. Bias (MD) and limits of agreement (LoA, MD±1.96*SDD) were plotted on the Bland-Altman plots. Mean absolute error (MAE) and mean absolute percentage error (MAPE) were calculated for HR and EE. Intraclass correlation coefficient (ICC) estimates were calculated for each set of data based on an average measures, absolute agreement, 2-way mixed-effects model.

Visual examination of the Bland-Altman plots was used to rule out systematic error; bias and LoA were used to assess for clinical applicability. ICC was calculated to determine the correlation between Apple Watch measurements and gold standard measurements. Limits for ICC were used as suggested by Fokkema et al [10]: an ICC >0.90 was considered excellent, 0.75 to 0.90 was good, 0.60 to 0.75 was moderate, and <0.60 was low.

For all statistical tests, the alpha level adopted for significance (2-tailed) was set at *P*<.05. All statistical analyses were performed using SPSS Statistics version 25 (IBM Corp).

## Results

### Patient Characteristics and Exercise Capacity

A total of 40 patients (32 male, 8 female) were included in this study. All patients had established cardiovascular disease: ischemic heart disease (28/40), valvular heart disease (9/40), and other type of heart disease (3/40). Further patient characteristics are depicted in Table 1. All participants performed the exercise test until exhaustion. Numeric test results are summarized in Table 2.

**Table 1:** Patient characteristics.

Characteristics			Value
Age in years, mean (SD)			61.9 (15.2)
Male gender, n (%)			32 (80)
Weight (kg), mean (SD)			79.0 (16.2)
Height (cm), mean (SD)			171.1 (9.3)
Body mass index (kg/m^2^), mean (SD)			27.0 (5.0)
**Cardiac disease type, n (%)**			
	Ischemic heart disease		28 (70)
	Valvular heart disease		9 (23)
	Other		3 (8)
**Cardiovascular risk factors, n (%)**			
	Family history of cardiovascular disease		20 (50)
	Hypertension		18 (45)
	Hypercholesterolemia		23 (58)
	Hypertriglyceridemia		10 (25)
	Overweight (body mass index ≥25)		27 (68)
	Obesity (body mass index ≥30)		9 (23)
	**Diabetes mellitus (total)**		8 (20)
		Diabetes mellitus (type 1)	1 (3)
		Diabetes mellitus (type 2)	7 (18)
	**Smoking (total)**		27 (68)
		Ex-smoker	26 (65)
		Current smoker	1 (3)
	Atrial fibrillation		5 (13)
**CPET^a^** **parameters**			
	CPET time (sec), mean (SD)		512 (194)
	VO_2_ peak^b^ (L/min), mean (SD)		1.72 (0.89)
	VO_2_ peak (mL/kg/min), mean (SD)		21.8 (11.6)
	Heart rate reserve (bpm), mean (SD)		56 (29)

^a^CPET: cardiopulmonary exercise test.

^b^VO_2_ peak: peak oxygen uptake.

### Heart Rate

**Figure 1:**
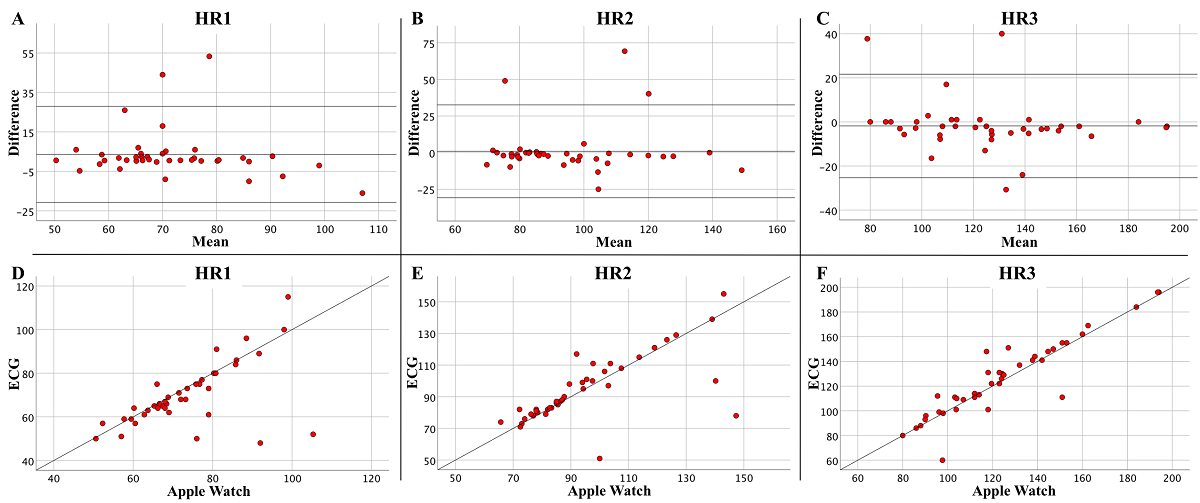
Heart rate (HR) measurements (bpm) by the Apple Watch are compared with gold standard electrocardiogram measurements for HRs at start (seated rest, HR1), middle (moderate intensity, HR2), and maximal performance level (HR3) of the cardiopulmonary exercise test.

SDD for HR1, HR2, and HR3 was 12.4, 16.2, and 12.0, respectively. Bias (ie, mean difference) and LoA were 3.61 (–20.74, 27.96) for HR1, 0.91 (–30.82, 32.63) for HR2, and –1.82 (–25.27, 21.63) for HR3. MAE was 6.34 for HR1, 7.55 for HR2, and 6.90 for HR3. MAPE was 10.69% for HR1, 9.20% for HR2, and 6.33% for HR3. The ICC was 0.729 (*P*<.001) for HR1, 0.828 (*P*<.001) for HR2, and 0.958 (*P*<.001) for HR3. Following the previously mentioned limits, this can be interpreted as a moderate correlation for HR1, a good correlation for HR2, and an excellent correlation for HR3. Bland-Altman plots and scatterplots comparing Apple Watch and ECG registration are depicted in Figure 1.

The Bland-Altman plots are depicted in A, B, and C and compare mean values on the x-axis ([Apple Watch + gold standard]/2) with the difference of the values on the y-axis (Apple Watch – gold standard). Bias and limits of agreement are depicted as horizontal lines. The plots depicted in D, E, and F directly compare values measured by the Apple Watch (x-axis) versus ECG measurements (y-axis). All plots show a good correlation of measurements without a systematic error.

**Table 2:** Sample size, correlation, and agreement between Apple Watch and reference methods for heart rate at start (seated rest, HR1), middle (moderate intensity, HR2), and maximal performance level (HR3), and energy expenditure (n=40).

Characteristics	HR1^a^ (bpm)	HR2^b^ (bpm	HR3^c^ (bpm)	Energy expenditure (kcal)
Gold standard measurement, mean (SD)	69.9 (14.5)	94.6 (20.6)	126.5 (30.9)	40.6 (32.4)
Gold standard measurement, standard error	2.30	3.26	4.88	6.49
SDD^d^, mean (SD)	3.61 (12.4)	0.91 (16.2)	–1.82 (12.0)	30.47 (17.5)
Upper LoA^e^	27.96	32.63	21.63	64.74
Lower LoA	–20.74	–30.82	–25.27	–3.80
MAE^f^	6.34	7.55	6.90	30.77
MAPE^g^ (%)	10.69	9.20	6.33	114.72
ICC^h^ (*P* value)	0.729 (<.001)	0.828 (<.001)	0.958 (<.001)	0.797 (<.001)

^a^HR1: heart rate, seated rest.

^b^HR2: heart rate, moderate intensity.

^c^HR3: heart rate, maximal performance level.

^d^SDD: standard deviation of difference.

^e^LoA: limits of agreement.

^f^MAE: mean absolute error.

^g^MAPE: mean absolute percentage error.

^h^ICC: intraclass correlation coefficient.

### Energy Expenditure

**Figure 2:**
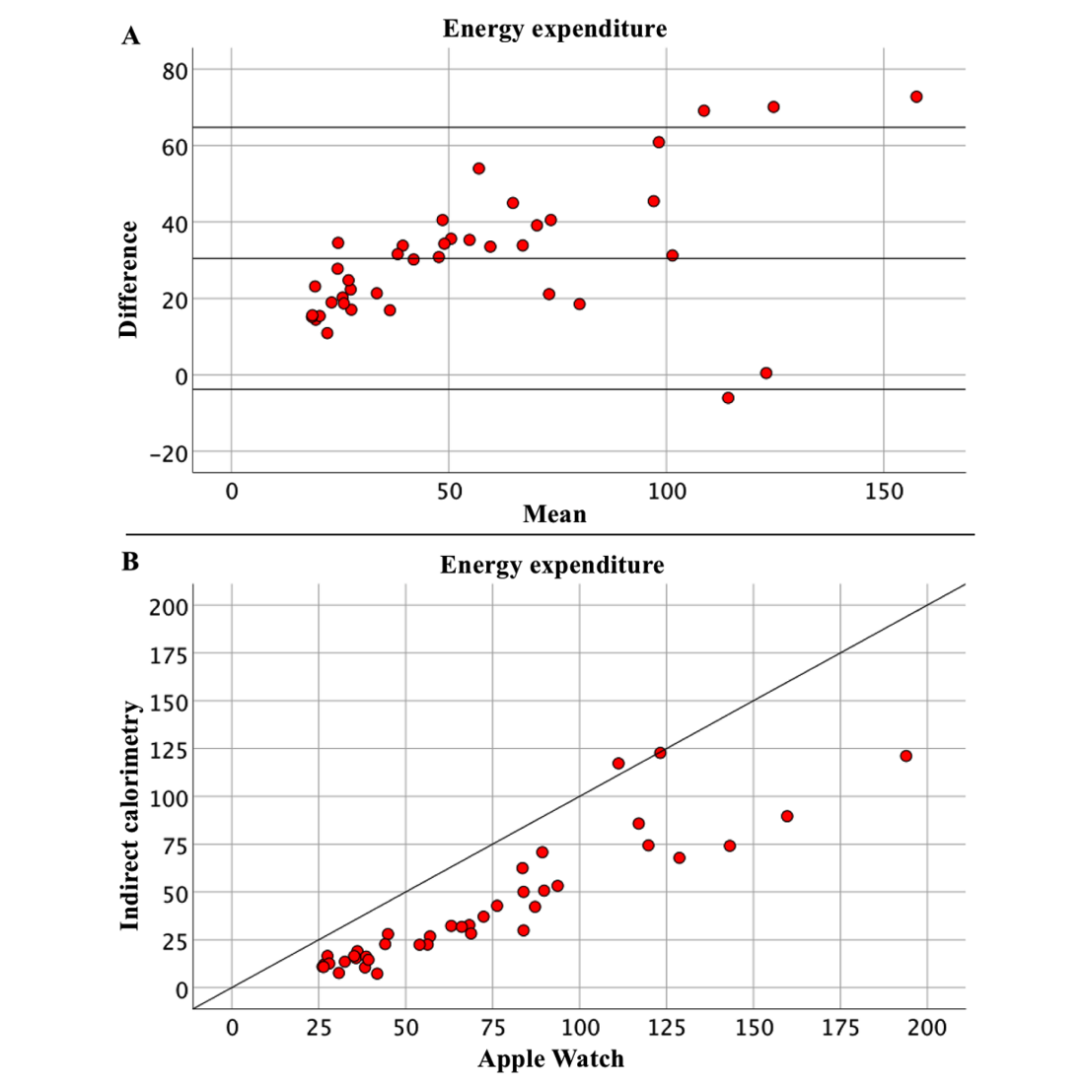
Energy expenditure (EE) measurement (kcal) by the Apple Watch is compared with gold standard indirect calorimetry. The Bland-Altman plot compares mean values on the x-axis ((Apple Watch + gold standard)/2) with the difference of values on the y-axis (Apple Watch – gold standard) (A). Bias and limits of agreement are depicted as horizontal lines. The plot depicted in part B directly compares values measured by the Apple Watch (x-axis) versus indirect calorimetry measurements (y-axis). A systematic error is seen with an overestimation of EE by the Apple Watch.

SDD for EE was 17.5. Bias and LoA were 30.47 (–3.80, 64.74). MAE was 30.77; MAPE was 114.72%. The ICC for EE was 0.797 (*P*<.001), which can be interpreted as a good correlation. Bland-Altman plot and a scatterplot directly comparing Apple Watch and indirect calorimetry are depicted in Figure 2. A systematic error is seen with an overestimation of EE by the Apple Watch.

## Discussion

### Principal Findings

For HR, accuracy, as evaluated by the SDD, was best at peak exercise intensity and lowest at moderate exercise intensity. ICC was highest at peak exercise intensity and lowest for resting HR. On the other hand, bias was largest for resting HR and smallest at moderate intensity. Bland-Altman plots and scatterplots show a good correlation of measurements without a systematic error. MAPE is highest at seated rest and lowest at maximal intensity. MAPE range is between 6.33% and 10.69%.

When relating these numbers to clinical practice and thus to actual HR measurement, the numbers for bias can be considered low (ie, no systematic error is made when measuring HR with the Apple Watch). The SDDs are within an acceptable range to be clinically relevant. MAPE values are considered low compared to EE values and compared to earlier studies.

Our results thus show good accuracy of HR measurements by the Apple Watch when compared to the gold standard ECG measurements when tested in patients with known heart disease.

For EE, SDD was 17.5, and bias was 30.47. The ICC is 0.797, which is considered good correlation. MAPE is 114.72%, which is high when compared to the MAPE range of HR measurements. The SDD is within an acceptable range for clinical practice. The bias, however, is quite large, meaning a systematic error with an average of 30.47 kcal per CPET test is made when using the Apple Watch for measuring calories compared to indirect calorimetry.

This systematic error is also seen when analyzing the scatterplot directly comparing the Apple Watch with indirect calorimetry: measurements of indirect calorimetry correlate with higher values measured by the Apple Watch. On the Bland-Altman plot, values are situated around a positive bias of 30.47 with almost all values being in the positive range.

It can thus be concluded that during CPET the Apple Watch systematically measures a higher value for EE than indirect calorimetry when measured in patients with known heart disease.

Studies comparing wrist-worn devices and in particular the Apple Watch with gold standard methods have already shown a good accuracy of HR measurement and a generally poor accuracy of EE measurement [4-7,11-13]. Similar ranges for MAPE for HR and EE were found in earlier studies [5,9]. Accuracy of EE measurement was found to vary depending on type of exercise and exercise intensity with a lower device error for running versus walking but a higher device error at higher levels of intensity for both running and walking [12]. In other studies, it was already shown that in healthy subjects the Apple Watch overestimated EE during cycling and resistance exercise [6].

Multiple studies aimed to validate commercially available devices for clinical practice, and Shcherbina et al state that there is an ongoing need to do so [12]. To our knowledge, this is the first study that evaluates accuracy of HR and EE monitoring by a wrist-worn device such as the Apple Watch in patients with proven cardiovascular disease.

In our study, it was shown that in patients with cardiovascular disease, the Apple Watch measures HR during exercise with clinically acceptable accuracy: there was no systematic error and bias was small compared to ranges of HR recommended in rehabilitation programs. If further studies confirm these results, it might be considered safe to incorporate the Apple Watch in HR-guided training programs in the setting of cardiac rehabilitation. At this moment, however, data remains uncertain, and although the wearable can be used to track activities and motivate patients, it is too early to recommend the Apple Watch for clinical usage in a cardiac rehabilitation setting.

EE measurements were not accurate, with a tendency of the Apple Watch to systematically overestimate EE during CPET testing. Caution should therefore be taken when using the Apple Watch in rehabilitation programs in which caloric balance is important (eg, weight loss programs in the setting of cardiac rehabilitation).

### Limitations

This study has limitations. HR was assessed in patients with known cardiac disease; this group was, however, a heterogeneous group with the majority of patients having ischemic or valvular heart disease. No subgroup with known arrhythmia was included. We therefore cannot state that accuracy of HR monitoring is good in all types of patients with known heart disease. Further studies are needed in patient groups with different types of cardiovascular disease to fully assess validity of the Apple Watch in these subgroups.

This study was nonrandomized. Due to the high proportion of included patients who suffered from ischemic heart disease, there is a male predominance of study participants (80%). Subgroup analysis showed no significant difference between male and female groups for mean difference. However, this analysis is prone to error due to small patient size. Shcherbina et al showed that the error rate for measurement in males was significantly higher than the error rate in females [12]. Further studies are needed to assess if there is indeed a difference in registration.

Further, exercise intensity was evaluated based on cycling resistance (test duration) only, by using a proportion of the maximally achieved resistance. Assessing ratings of perceived exertion would have added useful information.

EE was only assessed with data available through Apple general software. As mentioned in other studies [4], algorithms used to determine EE are not disclosed by the manufacturers. An independent study with transparent cooperation of manufacturers would be an interesting next step.

This study cannot distinguish between subgroups in which limitations inherent to PPG measurement are evident (eg, patients with darker skin tone, larger wrist circumference, higher BMI) [12]. During the CPET, the wrist was kept still while cycling, so no error should be expected from arm movement.

To increase comparability between standard measurements and Apple Watch measurements, it was decided to stop measurement at the exact moment the patient stopped the exercise. No measurements were thus performed in the resting phase after the CPET.

### Conclusion

Our results show that in patients with cardiovascular disease, the Apple Watch measures HR with clinically acceptable accuracy for 30 second averages of indoor cycling with the wrist kept stable. If confirmed, it might be considered safe to incorporate the Apple Watch in HR-guided training programs in the setting of cardiac rehabilitation. At this moment, however, it is too early to recommend the Apple Watch for cardiac rehabilitation. Also, the Apple Watch systematically overestimates EE in this group. Caution should therefore be taken when using the Apple Watch for measuring EE.

## Abbreviations

bpm: beats per minute
BMI: body mass index
CPET: cardiopulmonary exercise test
ECG: electrocardiogram
EE: energy expenditure
HR: heart rate
HRR: heart rate reserve
ICC: intraclass correlation coefficient
LoA: limits of agreement
MAE: mean absolute error
MAPE: mean absolute percentage error
MD: mean difference
PPG: photoplethysmography
SDD: standard deviation of difference
VCO_2_: carbon dioxide
VO_2_: oxygen uptake
